# Wolf Presence Disrupts Seasonal Variation in Hair Cortisol Among Free‐Ranging Beef Cattle

**DOI:** 10.1002/ece3.73431

**Published:** 2026-04-06

**Authors:** Christina M. Nord, Alexander J. Pritchard, Rosemary A. Blersch, Brenda McCowan, Jessica J. Vandeleest, Kenneth W. Tate, Tina L. Saitone

**Affiliations:** ^1^ Neuroscience and Behavior Unit, California National Primate Research Center University of California Davis Davis California USA; ^2^ Department of Population Health & Reproduction, School of Veterinary Medicine University of California Davis Davis California USA; ^3^ Department of Plant Sciences University of California Davis Davis California USA; ^4^ Department of Agricultural and Resource Economics University of California Davis Davis California USA

**Keywords:** beef cattle, California, gray wolf, hair cortisol, non‐consumptive effects

## Abstract

Understanding how predator reintroduction indirectly affects prey physiology remains poorly understood. We examined hair cortisol concentrations in 79 beef cattle from nine herds across California's Sierra Nevada, comparing herds exposed to the Lassen Wolf Pack with unexposed herds. We collected hair samples before and after summer grazing (*n* = 158 samples) and used Bayesian multilevel regression to test for effects of wolf presence. Wolf‐exposed herds showed a 58% reduction in temperature sensitivity compared to unexposed herds (where cortisol decreased 13.5% per 1°C), suggesting altered seasonal cortisol regulation. These preliminary results point toward a potential physiological mechanism by which predator reintroduction may indirectly affect prey, with implications for understanding how recovering carnivores could influence prey physiology, animal welfare, and productivity.

## Graphical abstract

Predators exert indirect effects on prey populations beyond direct predation, but how predator reintroduction restructures prey physiology remains poorly understood. Using hair cortisol analysis in cattle exposed versus unexposed to wolves, we found that wolf presence disrupted normal seasonal cortisol regulation patterns. These results reveal an important physiological mechanism by which predators can restructure prey ecological responses to environmental cues, with implications for understanding predator reintroduction dynamics.

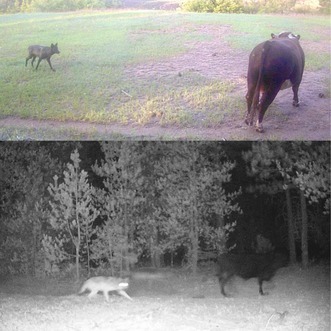

## 1 Introduction

Gray wolf reintroduction, dispersion, and natural recolonization across North America provide opportunities to understand how recovering predators restructure prey populations and communities, as well as knowledge essential for predicting ecosystem consequences of carnivore recovery. Most research focuses on direct predation effects; however, predators influence prey populations through non‐consumptive pathways—fear‐induced behavioral changes and physiological stress responses that alter foraging, reproduction, and survival independent of actual predation (Creel et al. 2009; Sheriff et al. 2011). These indirect effects can be equally or more important than direct predation in structuring prey populations and communities (Preisser et al. 2005; Creel and Christianson 2008).

Predator presence can impose indirect costs on prey species, including disrupted allostasis (McEwen and Wingfield 2003), with important implications for animal welfare and productivity (Steele et al. 2013). While these effects are well documented in wildlife (Creel et al. 2009; Sheriff et al. 2011) and in managed livestock settings (Munksgaard and Simonsen 1996; Bristow and Holmes 2007), there is a critical gap in our understanding of how free‐ranging animals from populations with no recent wolf exposure respond to large carnivore predators. Cattle in the Sierra Nevada represent a particularly relevant case study: wolves were extirpated from the region over a century ago, meaning the current cattle population lacks ecological memory of predator presence—that is, accumulated behavioral and physiological adaptations that persist in populations with continuous predator exposure. Thus, these cattle may lack the physiological responses typically evolved under predation risk (Kovacs et al. 2016; Schweiger et al. 2019). Livestock grazing is prevalent across predator habitat in western North America, making livestock–predator interactions an important ecological coexistence challenge. Yet little is known about the longer‐term physiological costs incurred when naïve animals must cope with new—and real—predator threats (Ewacha 2016; Cooke 2017; Valerio et al. 2021; Rafael 2022). Wolves actively prey on livestock in western North America (Steele et al. 2013; Dellinger et al. 2021; California Department of Fish and Wildlife 2025; Martin et al. 2025), creating genuine predation risk; however, most ecological and welfare research has focused on either short‐term experimental simulations of predator risk (Apfelbach et al. 2005; Cooke et al. 2013; Cooke 2017) or wildlife already living with predators (Valerio et al. 2021; Rafael 2022) rather than on longer‐term physiological costs in livestock populations transitioning to sympatry with recovering predators.

Cortisol plays a key role in energy regulation but can be elevated in response to challenges or stressors, resulting in deleterious effects when chronically elevated (Sapolsky et al. 2000). In livestock, sustained increases in cortisol have been linked to compromised health and productivity (Kluever et al. 2009; Laporte et al. 2010; Cooke et al. 2013; Ramler et al. 2014). Hair cortisol provides a non‐invasive, integrative biomarker of chronic stress physiology, as cortisol is incorporated into growing hair over weeks to months (Greff et al. 2019; Heimbürge et al. 2020; Van Eerdenburg et al. 2021). This approach has been used to document stress in managed livestock settings and in free‐ranging wildlife (Macbeth et al. 2010; Ewacha 2016), but to our knowledge has not been applied to free‐ranging livestock populations experiencing natural predator reintroduction.

**FIGURE 1:**
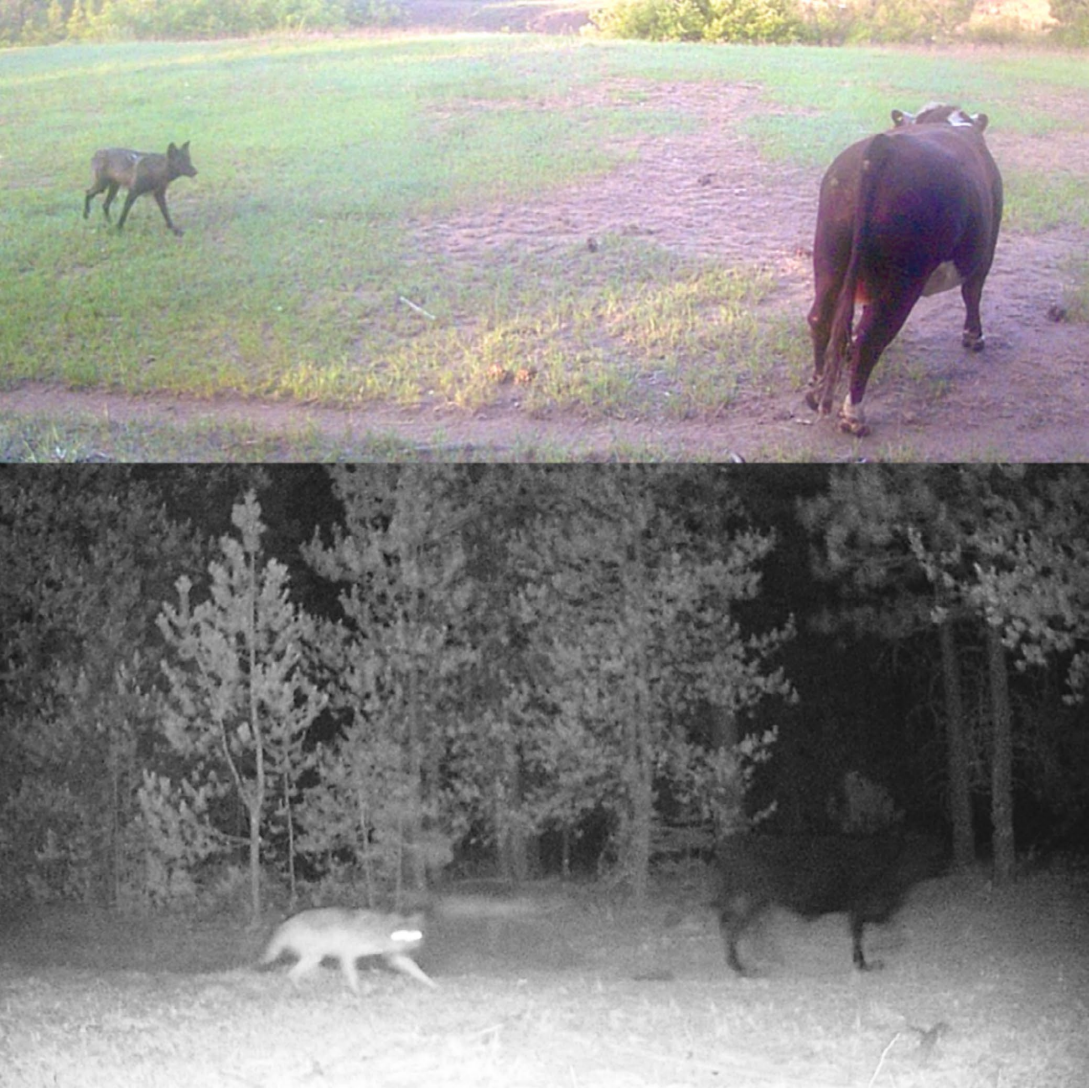
Cattle encounter gray wolves ( *Canis lupus* ) in the study area in California's Sierra Nevada. Top: A *B. taurus* bull and a member of the Lassen Wolf Pack ( *Canis lupus* ). Bottom: A first calf heifer ( *B. taurus* ) and a member of the Lassen Wolf Pack. Image credit: K.W. Tate and T.L. Saitone.

Here, we present the first longitudinal study of hair cortisol concentrations in free‐ranging beef cattle of mixed European ( *Bos taurus* ) and European × Zebu ( *B. taurus* × *B. indicus* ) breeding exposed to gray wolves ( *Canis lupus* ) (Figure 1). We sampled hair cortisol from 79 cattle across nine herds, with some herds experiencing wolf presence during summer grazing while others did not. This comparative design allowed us to evaluate how predator exposure interacts with seasonal environmental variation. We hypothesized that cattle with no prior wolf exposure would exhibit physiological dysregulation when moved to summer grazing lands where wolves were present, compared to cattle in wolf‐free areas (Romero et al. 2009; Valerio 2019).

Temperature is a key environmental cue affecting cortisol expression in vertebrates, with warm seasons typically associated with lower glucocorticoid levels (Alvarez and Johnson 1973; Lee et al. 1976). Transitions between grazing conditions, such as differing forage quality and environment (Comin et al. 2011; Ghassemi Nejad et al. 2021), can influence cortisol levels. Assuming that wolf presence could disrupt allostasis, we predicted that this disruption would manifest as altered responsiveness to environmental cues—specifically, a disrupted temperature–cortisol relationship in wolf‐exposed herds compared to the normal seasonal decline observed in unexposed herds (Romero et al. 2009; Valerio 2019; Valerio et al. 2021; Romero and Beattie 2022)—relative to wolf‐free herds. Importantly, we did not predict a simple elevation in mean cortisol concentrations; chronic stress can manifest as altered regulatory dynamics rather than elevated baselines (Romero and Beattie 2022), and predator presence may specifically interfere with the physiological adjustments prey normally make in response to predictable environmental variation. We also examined seasonal variation, as temperature affects cortisol expression (Romero 2002; De Bruijn and Romero 2018; Pritchard et al. 2025).

By comparing wolf‐exposed and wolf‐free herds, our study provides a preliminary examination of the physiological mechanisms of non‐consumptive predator effects during carnivore reintroduction with broad implications for predator–prey ecology and the management of shared landscapes in the context of carnivore recovery.

## 2 Study Area

This study was conducted between May and November 2022, across 780,000 acres of contiguous United States Forest Service (USFS) allotments and privately‐owned rangelands and timberlands spanning western Lassen and northwestern Plumas Counties, California, United States of America. We enrolled five rangeland cattle operators, each managing one or more grazing units (USFS allotments and/or private grazing leases) within the study area. Privately‐owned land in the region is intermixed with USFS allotments, with each herd grazing 40–121 km^2^ at stocking rates between 600 and 3200 animal unit months (AUMs). Collectively, these operators managed roughly 2400 commercial cow‐calf pairs that used these lands during the grazing season.

Across allotments there are predators including bears, mountain lions, coyotes, and bobcats. Importantly, a portion of the study area includes the documented home range of the Lassen Wolf Pack. The distribution of grazing allotments enrolled in the study represents a gradient of wolf exposure—from areas with high wolf–cattle interaction due to denning and rendezvous sites to areas with no documented wolf presence (California Department of Fish and Wildlife 2023, 2025). The region has a Mediterranean climate with cool, moist winters and warm, dry summers. Temperatures peak in July (mean: 19.5°C; mean daily minimum: 9.0°C; mean daily maximum: 30.1°C) and reach lows in December (mean: −0.2°C; mean daily minimum: −5.6°C; mean daily maximum: 5.2°C) (National Weather Service, n.d.). The landscape includes riparian areas, upland meadows, sagebrush, aspen stands, and coniferous forests. Broad open meadows, separated by forested buttes, provide the primary livestock forage. Although riparian and aspen habitats cover only ~2% of the area, they contribute significantly to landscape diversity and are prioritized for conservation and enhancement.

## 3 Methods

### 3.1 Herds

We followed nine commercial cow herds that operate under typical western United States' cow–calf systems. Wolf‐affected herds were enrolled based on the historic geographic home range of the Lassen Wolf Pack. Herds unexposed to wolves were enrolled to minimize geographic, climate, ecological, and management differences relative to wolf‐affected herds with attempts to match non‐wolf exposed herds based on proximity to wolf‐affected herds and similarities in elevation, habitat type, and management practices. In June, cattle are moved from low‐elevation (100–1500 ft) privately‐owned or leased annual grassland to the higher‐elevation (5000–7500 ft) USFS summer grazing allotments, before returning to winter range in October. Herd sizes ranged from 400 to 500 head of English bred cattle (e.g., Angus, Angus‐cross, Herford) or Beefmaster cattle (a breed created by crossing Hereford, shorthorn, and Brahman) (Table 1). Of these, six herds (Herds A–F) grazed in areas with no documented wolf activity. Three herds (Herds G–I) grazed in areas with regular activity by the Lassen Wolf Pack (see subsection below) and experienced wolf‐related impacts, including confirmed depredations (Kovacs et al. 2016; Dellinger et al. 2021; California Department of Fish and Wildlife 2023, 2025).

**TABLE 1:** Herd characteristics, sampling details, and environmental data for beef cattle in California's Sierra Nevada, 2022.^a^

Herd	Wolf presence	Herd population size	Cattle sampled (*n*. paired winter to summer)	Breed(s) and coat color(s)	Winter sample date	Summer pasture arrival date	Summer pasture departure date	Summer sample date	Winter mean daily minimum temperature (°C)^b^	Summer mean daily minimum temperature (°C)^b^
A	No	150	13	Black English	18 May 2022	4 June 2022	14 October 2022	24 October 2022	8.69 (−1.1 to 17.0, 3.59)	10.58 (−1.2 to 22.7, 4.67)
B	No	103	9	Red, Yellow Beef Master, Black English	13 June 2022	15 June 2022	22 September 2022	30 September 2022	11.53 (1.0 to 24.0, 4.92)	12.34 (0.9 to 20.7, 4.09)
C	No	100	7	Black, Red English	5 June 2022	7 June 2022	12 October 2022	16 November 2022	10.41 (−1.9 to 22.5, 5.08)	11.53 (0.4 to 20.6, 4.31)
D	No	102	8	Red, Yellow Beef Master, Black English	8 June, 13 June 2022	14 June 2022	31 August 2022	30 September, 1 October 2022	11.53 (1.0 to 24.0, 4.92)	13.25 (1.1 to 25.8, 4.88)
E	No	200	11	Red, Yellow Beef Master	1 June 2022	1 June 2022	8 October 2022	7 October, 8 October 2022	8.33 (−3.8 to 19.9, 4.71)	10.11 (0.3 to 20.4, 4.28)
F	No	95	5	Black English	11 May 2022	13 June 2022	11 November 2022	16 November 2022	7.59 (−1.9 to 18.7, 4.31)	12.19 (−8.4 to 24.2, 6.17)
G	Yes	104	7	Red, Yellow Beef Master, Black English	13 June 2022	9 June 2022	22 September 2022	1 October, 9 October 2022	10.74 (−2.1 to 22.3, 5.05)	13.44 (2.8 to 21.4, 4.10)
H	Yes	100	2	Red, Yellow Beef Master	8 June 2022	9 June 2022	22 September 2022	1 October, 9 October 2022	10.55 (−2.1 to 22.3, 5.07)	13.59 (2.8 to 21.4, 3.98)
I	Yes	201	17	Black, Red English	14 May 2022	16 May 2022	3 October 2022	31 October 2022	6.19 (−3.0 to 15.9, 3.62)	10.96 (−1.2 to 21.2, 4.74)
Total^c^		1095	79						9.51 (−3.8 to 24.0, 4.93)	11.88 (−8.4 to 25.8, 4.86)

Abbreviation: SD, standard deviation.

^a^ Wolf presence indicates whether herds were exposed to the Lassen Wolf Pack during the 2022 grazing season.

^b^ Temperature data presented as mean (range, SD).

^c^ Totals row shows combined herd population size, total cattle sampled, and mean temperatures across all herds.

These nine focal herds were otherwise comparable in breeding, herd management, range conditions, and climate exposure, allowing for a robust comparison of outcomes under differing levels of wolf presence. All herds grazed on similar mixed‐conifer forest and montane meadow habitats during summer, at comparable elevations and stocking densities, with access to similar forage types and water resources. Non‐wolf predators—including bears, mountain lions, coyotes, and bobcats—are present throughout all study areas, with no documented or intuited reason to expect systematic differences in non‐wolf predation risk between wolf‐exposed and unexposed herds. We assessed potential sampling bias using the STRANGE framework (Webster and Rutz 2020), which evaluates whether study animals are representative across dimensions including Social background, Trappability, Rearing history, Acclimation, Natural changes, Genetic makeup, and Experience. Our cattle sample exhibited moderate STRANGEness: though limited to mature cows from working ranches in a single region, all ranchers in the region enrolled, and the animals experienced typical grazing conditions and management practices for this production system. These qualities suggest reasonable generalizability to similar extensive beef production systems.

### 3.2 Lassen Wolf Pack

The Lassen pack, California's second documented wolf pack, occupies approximately 332 mile^2^ across western Lassen County and northern Plumas County, including portions of the Lassen National Forest. Since producing their first litter of four pups in 2017, the pack has successfully reproduced annually with litter sizes ranging from 2 to 9 pups per year through 2022 (Dellinger et al. 2021). During our study, the pack consisted of at least a dozen members (Petri 2022; Martin et al. 2025).

### 3.3 Biological Samples

We batch‐collected hair samples during each study period from 79 animals in the nine study herds (Table 1), with each animal sampled twice to create paired longitudinal observations. We sampled individuals before their move to summer grazing lands—where none of the herds were exposed to wolves—and again upon their return to winter grasslands, after the potential for wolf exposure during summer grazing (mean = 20.2 weeks ±3.9 SD weeks; range: 15.6–27.0 weeks; Table 1). This paired design allowed us to use the winter sampling period as a pre‐exposure baseline, with post‐summer sampling capturing any effects of differential wolf exposure. Cows were selected without a formal randomization protocol, but with no specific selection criteria. Calves and yearling cattle were not sampled to reduce potential glucocorticoid variation related to age.

We used a shave re‐shave sampling method and followed the standard protocol (Meyer et al. 2014; Vandeleest et al. 2019; Heimbürge et al. 2020; Pritchard et al. 2023), collecting approximately 3 cm of hair growth during the winter and summer sampling periods. During initial winter hair collection, we clipped hair from the tail tip (i.e., tail switch) as close to the skin as possible, using surgical clipper blades, and retained the 3 cm closest to the skin for analysis. The summer sample was harvested from the same region and in the same manner.

We collected a total of 158 hair samples from 79 animals (26 of which were wolf‐exposed) across the 2 study periods (one sample per subject per study period). Population demographics and herd summary statistics are provided in Table 1.

### 3.4 Average Minimum Temperatures

Daily minimum temperature data were obtained for the geographic centroid of each herd's grazing area using Open‐Meteo (Zippenfenig 2025), which provides gridded meteorological data interpolated to specific coordinates (Zippenfenig 2025). For the first sample, we used the same time interval measured backward from the collection date to ensure similar temperature exposures across all samples, accounting for hair growth rates given that each sample was the same length. These values were averaged across each study period to calculate the seasonal mean minimum temperature for each herd's grazing area.

### 3.5 Cortisol Assay

We extracted cortisol from the 3‐cm hair samples and assayed the samples using enzyme immunoassays (Salimetrics, Carlsbad, CA, USA), as previously validated (Meyer et al. 2014; Vandeleest et al. 2019; Heimbürge et al. 2020). We confirmed assay validity for cattle hair samples using parallelism and spike‐and‐recovery tests. Samples met acceptance criteria of parallel displacement curves with correlation coefficients *r* ≥ 0.95 and hormone recovery within 90%–110% of spiked concentrations. Using kit‐provided high‐ and low‐quality controls, interassay CVs averaged 13.02% ± 1.21 SD. We evaluated whether inter‐assay variability influenced our results by comparing models with and without assay plate as a random effect. Model comparison using leave‐one‐out cross‐validation indicated that including plate did not improve predictive accuracy relative to our selected model (ΔELPD = 0.7, SE = 2.3), so plate was excluded as a random effect. Retained samples had a mean intraassay CV of 1.27% ± 1.17 SD.

### 3.6 Statistical Analyses

We used a directed acyclic graph (DAG), generated using the daggity package in R (Textor et al. 2011), to formalize our assumptions about the causal relationship between wolf presence and cortisol concentrations and to identify the minimal sufficient adjustment set for unbiased estimation (Pearl 2009). A key advantage of the DAG framework is that it incorporates all variables believed to be relevant to the causal structure—including variables that were not or could not be measured—allowing us to evaluate whether our measured variables are sufficient to test our predictions while avoiding confounds. Our DAG identified potential confounding by animal location, which is structured by herd and season, and clarified that temperature may act as a mediator of seasonal effects. Based on the DAG, we included herd as a grouping variable to account for location‐level clustering and breed and coat color as an individual‐level predictor of cortisol. Season (study period) was included as a predictor and in interactions with wolf presence and temperature to allow us to test hypotheses about temporal variation in exposure effects. Variables appearing in the DAG but not in the final model either lie on causal pathways (and thus should not be conditioned on to avoid blocking the effect of interest) or are d‐separated from the exposure‐outcome—meaning they are conditionally independent of the outcome given our adjustment set and therefore do not require explicit inclusion in the model. Details and the full DAG are provided in the Supporting Information (Figure S1). Because herds graze in spatially fixed locations, temperature and herd identity are partially correlated. We addressed this by including herd as a random effect (capturing all stable herd‐level characteristics) and included temperature as a time‐varying predictor. This allowed us to estimate temperature effects from within‐herd variation across seasons while accounting for baseline differences between herds.

All analyses were conducted within a Bayesian framework using multilevel regression models (Gelman and Shalizi 2012) via the brms package in *R* (Bürkner 2017a, 2017b, 2019). All $\hat{R}$ values were equal to 1.0, indicating model convergence, and effective sample sizes (ESS) were within the acceptable range (Gelman and Shirley 2011). Model performance was evaluated using posterior predictive checks (pp_check; Bürkner 2017b; Figures S2–S4). We assessed collinearity among predictors using the pairs function in R (Bürkner 2017b) and found no strong evidence for multivariate correlations (Figure S5).

Plots were generated using ggplot2 (Wickham 2009) and the nord color palette (Kaupp 2018). We report 95% credible intervals (CI) in both tables and figures (McElreath 2016).

#### 3.6.1 Hair Cortisol Values in Relation to Study Period and Wolf Exposure

To determine if hair cortisol values were impacted by wolf presence, we constructed a multilevel regression model with a lognormal distribution. We included wolf presence (yes/no), study period (winter, summer), scaled average daily minimum temperature (mean = 0, SD = 1), and a breed–coat color variable as predictors. Animal ID was nested under herd as a grouping variable. Breed and coat color were combined into a single variable because they were not evenly distributed across herds and study periods. We assigned weakly informative priors (normal(0, 1)) to all predictor variables, as well as the model intercept. These priors reflect our assumption that large effect sizes are unlikely, but possible, and help stabilize estimation in the absence of strong prior knowledge. While our model allowed us to examine temperature‐wolf interactions, wolf presence was limited to three sites, resulting in relatively limited data coverage across the temperature gradient (Table 1).

**FIGURE 2:**
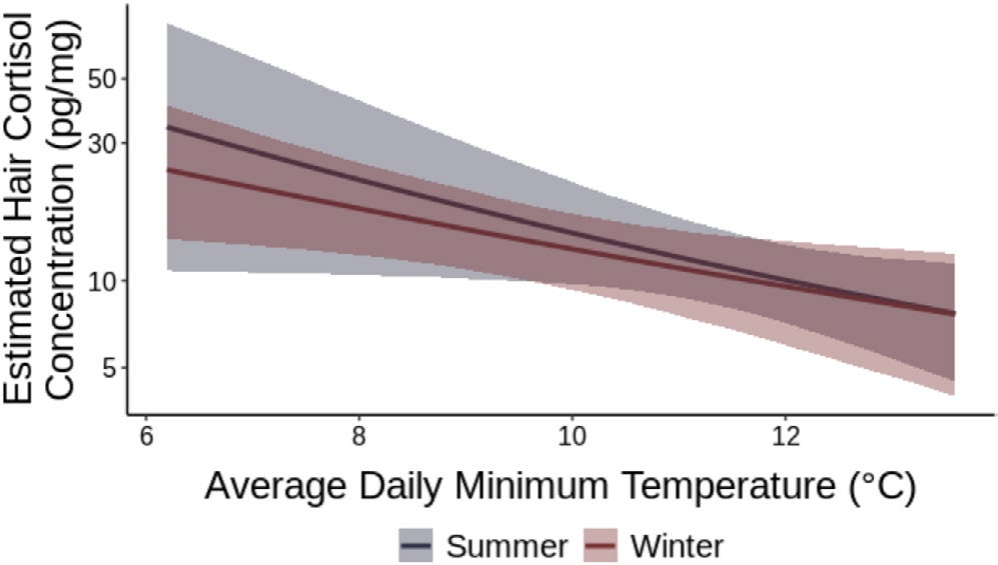
Population‐level hair cortisol predictions across temperature for winter (blue) and summer (red) study periods. Lines show posterior means; ribbons show 95% credible intervals. Predictions for unexposed cattle (Black English breed). Cortisol on log scale.

To examine model outcomes, we used posterior predictions from our Bayesian hierarchical model that included both fixed and random effects. To examine population‐level temperature effects (Figure 2), we generated predictions using the fitted() function in brms with random effects excluded (re_formula = NA) to focus on the average temperature–cortisol relationship across the population. We held wolf exposure and breed/color constant (no wolf impact, Black English) and varied temperature and study period across an extended temperature gradient. Visualizations were restricted to observed temperature ranges for each study period (winter: 5°C–13°C; summer: 9°C–15°C) to avoid extrapolation beyond our data.

**FIGURE 3:**
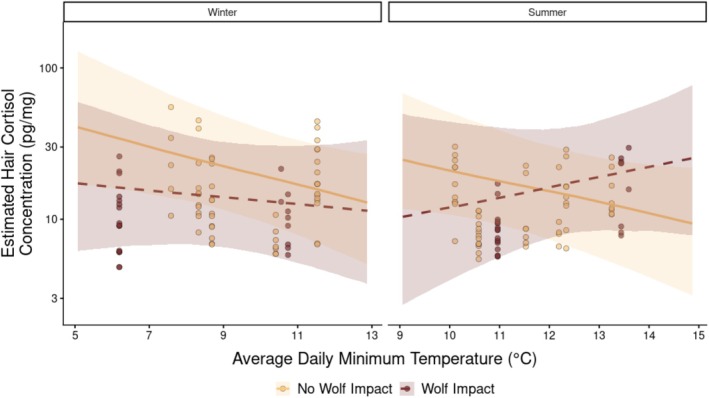
Conditional effects of temperature on cortisol by study period and wolf exposure. Points: Observed data. Lines: Model predictions with 95% credible intervals (ribbons). Yellow/solid: Unexposed herds. Red/dashed: Wolf‐exposed herds. Temperatures restricted to observed ranges; cortisol on log scale. Estimates for Black English breed.

We interpreted the three‐way interaction between study period, wolf exposure, and temperature at two hierarchical levels that allowed us to identify population‐level patterns and validate whether individual herds follow them. First, we generated conditional effects to visualize the population‐level interaction (Figure 3). Because this interaction involves categorical variables, it cannot be meaningfully interpreted from parameter estimates alone, which depend on reference category choices. Conditional effects provide population‐level predictions averaged over random effects, showing how the temperature–cortisol relationship varies by wolf exposure and season. We generated conditional effects using the conditional_effects() function in brms for wolf exposure (wolf impact vs. no wolf impact) and study period (winter vs. summer) across the temperature gradient. Visualizations were restricted to observed temperature ranges for each study period (winter: 5°C–13°C; summer: 9°C–15°C).

**FIGURE 4:**
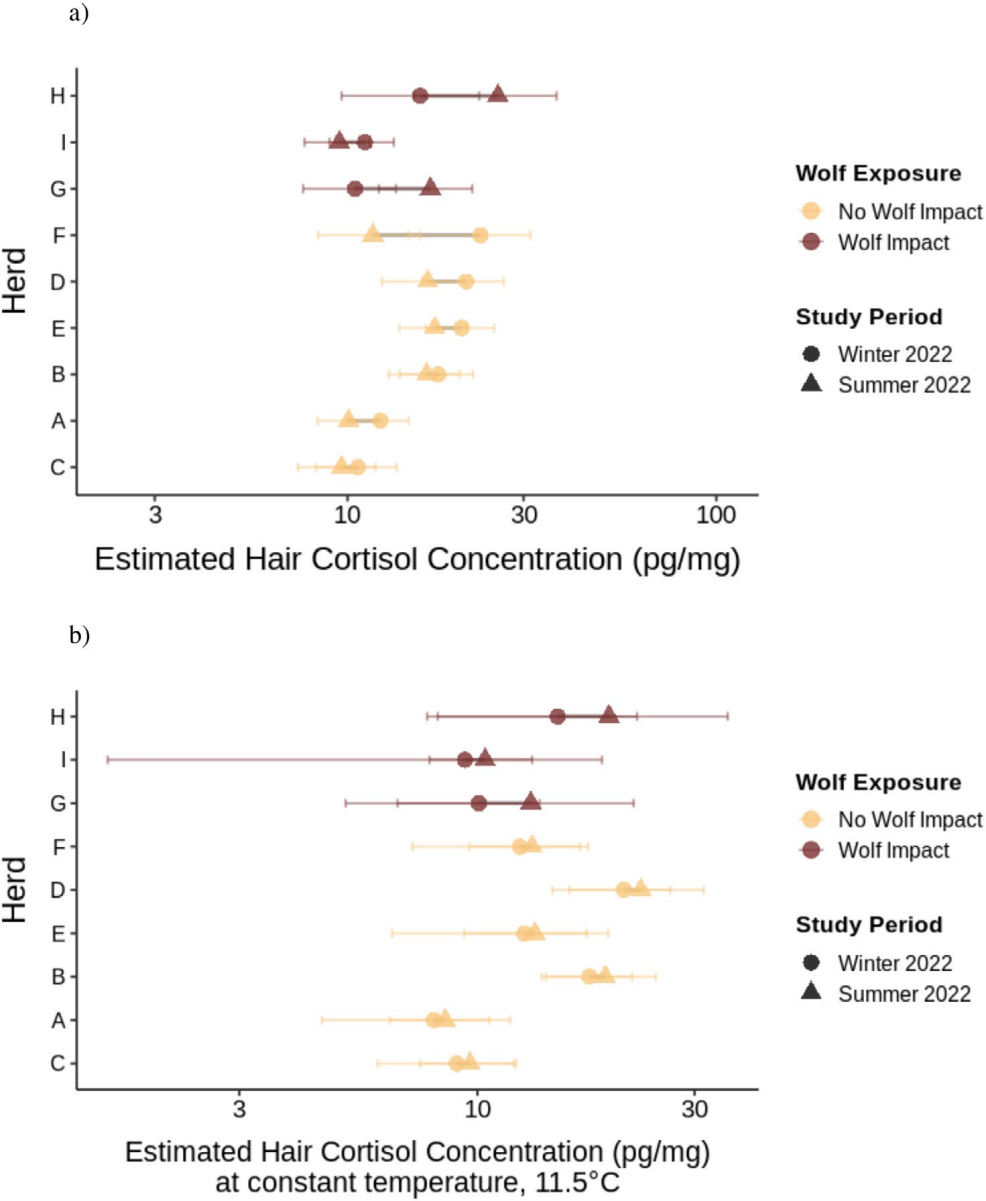
Herd‐specific winter‐to‐summer cortisol changes. Points represent posterior means of predicted cortisol concentrations (pg/mg) back‐transformed from the log‐normal model; error bars show 95% credible intervals. Circles: Winter; triangles: Summer. Lines connect time points for each herd. Yellow: Unexposed herds (A: *n* = 13, B: *n* = 9, C: *n* = 7, D: *n* = 8, E: *n* = 11, F: *n* = 5); red: Wolf‐exposed (G: *n* = 7, H: *n* = 2, I: *n* = 17). (a) Predictions at observed temperatures for each herd‐period. (b) Predictions at constant temperature (11.5°C) to isolate temporal effects. Cortisol displayed on log scale.

Second, because herd is both a random effect in our model and our unit of wolf exposure, we examined herd‐specific posterior predictions (Figure 4) to determine whether individual herds followed the population‐level pattern. To capture herd‐level variation, we used the fitted() function with random effects included (default re_formula = NULL). Unlike the population‐level predictions in Figure 2, these fitted values incorporate both population‐level effects (wolf exposure, study period, temperature, breed/color) and group‐level effects (random intercepts for herd and animal ID nested within herd). For each herd‐study period combination, we obtained posterior draws for all observations, averaged those draws within each herd‐period combination, then calculated means and 95% credible intervals from the distribution of averaged draws. This approach provides herd‐specific cortisol estimates that account for individual herd characteristics and allow us to assess whether the population‐level patterns are consistent at the level where wolf exposure occurs (Figure 4a). To isolate the effects of study period and wolf exposure independent of temperature variation, we repeated the analysis with temperature held constant at its maximum observed value across both study periods (approximately 11.5°C, or 0.596 after scaling; Figure 4b). This constant‐temperature comparison removes confounding by temperature differences between seasons and clarifies whether cortisol trajectories differ by wolf exposure beyond temperature‐driven changes. We present model results as summary statistics (Table 2) including posterior means, 95% CIs, and conditional and marginal *R* ^2^ values estimated using the bayes_R2 function (Gelman et al. 2019).

**TABLE 2:** Posterior estimates of hair cortisol concentrations (linear scale) from Bayesian multilevel regression model of beef cattle in California's Sierra Nevada, 2022.^a^ ^,^ ^b^

Effect	Parameter	Estimate	Estimated error	Lower 95% CI	Upper 95% CI	PD (%)
Population‐level effects	Intercept (no wolf impact, winter, Red Beef Master)	2.83	0.20	2.41	3.20	100.00
	Wolf impact	−0.34	0.32	−0.96	0.35	86.70
	Summer study period (SP)	0.11	0.18	−0.25	0.46	72.54
	Average daily minimum temperature	−0.32	0.16	−0.64	−0.03	98.51
	Yellow Beef Master	0.00	0.14	−0.27	0.29	50.01
	Black English	−0.43	0.14	−0.70	−0.15	99.76
	Red English	0.20	0.24	−0.26	0.69	79.14
	Wolf impact × Summer SP	−0.11	0.50	−1.11	0.84	58.74
	Wolf impact × Average daily minimum temperature	0.20	0.23	−0.24	0.68	80.83
	Summer SP × Average daily minimum temperature	−0.05	0.15	−0.34	0.24	64.86
	Wolf impact × Summer SP × Average daily minimum temperature	0.47	0.25	−0.02	0.97	96.96
Group‐level effects	SD(Herd)	0.38	0.18	0.12	0.84	100.00
	SD(Herd: Animal ID)	0.12	0.07	0.01	0.26	100.00

Abbreviations: CI, credible interval; PD, probability of direction.

^a^ Wolf impact relative to no wolf impact; study period relative to winter; breed and coat color relative to Red Beef Master. Estimates on linear scale.

^b^ *N* = 154 samples. *R* ^2^conditional = 0.38 (95% CI: 0.24–0.52); *R* ^2^marginal = 0.30 (95% CI: 0.15–0.46).

To quantify specific comparisons and provide formal contrasts, we calculated estimated marginal means (emmeans) using the emmeans package in R. We estimated marginal means for all combinations of wolf exposure, study period, and temperature at 3 standardized levels: −1 SD (cooler), mean, and + 1 SD (warmer) temperatures (Table S1). This allowed us to quantify the direction and magnitude of temperature–cortisol relationships within each group and study period, and to formally test whether these relationships differed between wolf‐exposed and unexposed herds.

## 4 Results

Based on the reference groups, hair cortisol concentration decreased as average daily minimum temperature increased (*β* = −0.32, 95% CI [−0.64, −0.03]; **Figure** **2**, **Table** **2**). For every 1°C increase in minimum temperature, cortisol decreased to ~86.5% of its previous value—a ~13.5% reduction. Hair cortisol concentration varied substantially by herd membership (SD = 0.38, 95% CI [0.12, 0.84]) and by individual animal within herd (SD = 0.12, 95% CI [0.01, 0.26]) (**Table** **2**). Wolf‐exposed and unexposed herds experienced broadly overlapping temperature ranges during both study periods (Table 1; winter: 6.2°C–11.5°C vs. 7.6°C–11.5°C; summer: 11.0°C–13.6°C vs. 10.1°C–13.3°C), indicating that wolf exposure status was not systematically confounded with temperature.

Hair cortisol concentrations varied among herds at baseline (random effect SD = 0.38, 95% CI [0.12, 0.84]; Table 2), likely reflecting herd‐level differences in variables such as genetics, management, or local conditions. This between‐herd variation is captured by random intercepts and does not affect inference about how cortisol‐temperature relationships differ by wolf exposure and season. Among wolf‐exposed herds, Herd I (*n* = 17) showed a slight decrease in cortisol at observed temperatures, while Herds G (*n* = 7) and H (*n* = 2) showed increases. This heterogeneity warrants caution: the clearest patterns emerged in herds with smaller samples, and the largest wolf‐exposed herd showed a weaker effect. However, at constant temperature (Figure 4b), all three wolf‐exposed herds showed increased or maintained cortisol.

### 4.1 Hair Cortisol Concentration and Average Daily Minimum Temperature: Is There a Relationship Between Hair Cortisol and Wolf Presence?

At the population level, we found evidence of a three‐way interaction between wolf exposure, study period, and average daily minimum temperature (*β* = 0.47, 95% CI [−0.02, 0.97], probability of direction = 96.96%; Table 2), though the magnitude of this effect was uncertain (Figure 3). During the winter study period, hair cortisol concentrations decreased slightly as average daily minimum temperature increased. However, during the summer study period, this relationship varied by wolf exposure: in unexposed herds, cortisol concentrations continued to decrease with increasing temperatures, whereas in wolf‐exposed herds, cortisol concentrations increased. Stated simply, we found evidence that wolf‐impact could moderate the relationship between cortisol and temperature—albeit with limited temperature sampling events per season. Even so, for herds exposed to wolves during the summer study period, the effect of increasing minimum temperature on cortisol was 58% higher than unexposed herds. Thus, these results suggest that wolf exposure altered the seasonal trajectory of hair cortisol.

To further understand these patterns, we estimated marginal means at cooler (−1 SD, ~8.3°C), mean, and warmer (+1 SD, ~12.3°C) temperatures. In summer, wolf‐exposed herds showed cortisol increasing from 9.3 pg/mg (95% HPD [0.6, 35.2]) at cooler temperatures to 16.9 pg/mg (95% HPD [9.8, 28.0]) at warmer temperatures. In contrast, unexposed herds decreased from 28.7 pg/mg (95% HPD [11.4, 59.0]) to 14.0 pg/mg (95% HPD [8.5, 19.4]). Formal contrasts of these temperature slopes showed uncertainty in the magnitude of this difference (slope difference = 10.0 pg/mg, 95% HPD [−2.60, 23.9]) meaning we cannot precisely quantify these distinctions.

Because our research question concerns how individual herds respond to wolf exposure, and herd is both a random effect and our unit of exposure, we examined herd‐specific posterior predictions that incorporate both fixed and random effects (Figure 4). Sample sizes varied substantially across herds, with wolf‐exposed herds contributing 17 (Herd I), 7 (Herd G), and 2 (Herd H) individuals respectively (Table 1); this heterogeneity warrants caution when interpreting herd‐specific patterns, particularly for Herd H. Using observed temperatures for each herd, we found that hair cortisol concentrations increased across study periods in two of the three wolf‐exposed, while concentrations decreased in all six unexposed herds (Figure 4a). To isolate temporal changes while accounting for temperature differences between study periods, we also evaluated posterior predictions holding temperature constant at 11.5°C, the highest value observed in both study periods (Figure 4b). Under constant‐temperature conditions, the pattern remained, albeit with high uncertainty: wolf‐exposed herds showed increases or maintenance of cortisol concentrations, while unexposed herds remained consistent.

## 5 Discussion

During summer, wolf‐exposed herds showed an inverted temperature–cortisol relationship, with concentrations increasing as temperatures rose, whereas unexposed herds showed the expected seasonal decline. While formal contrasts of these slopes reveal magnitude uncertainty, the directional pattern was observed in two of three individual wolf‐exposed herds at observed temperatures, and in all three wolf‐exposed herds when temperature was held constant. Further investigation is warranted to assess the consistency of this directional pattern across herds and its biological plausibility.

These findings suggest a potential mechanism of non‐consumptive predator effects: wolf presence may disrupt the physiological regulation that prey normally use to respond to environmental variation. In most systems, prey modulate cortisol seasonally in response to predictable environmental cues, adjusting metabolism and behavior accordingly (Romero 2002; De Bruijn and Romero 2018). The directional pattern we observed—where wolf‐exposed herds showed disrupted temperature‐dependent cortisol relationships—suggests that predators may restructure prey physiology in important ways, not simply by elevating absolute stress hormone concentrations, but potentially by altering the normal linkage between environmental cues and physiological responses. While theory and controlled experiments have shown that environmental cues associated with predation risk can restructure prey physiological state and stress responses (McNamara et al. 2005; Hawlena and Schmitz 2010), whether predators disrupt normal seasonal physiological modulation in free‐ranging prey remains poorly understood. If confirmed in larger samples, our finding would provide preliminary field evidence that predators may alter how prey physiologically respond to environmental variation (Batabyal 2023), a mechanism with significant implications for understanding non‐consumptive predator effects on prey ecology.

The observed temperature–cortisol relationship in wolf‐exposed herds, if confirmed with larger samples, would indicate changes in normal physiological patterns. While we did not find universally elevated cortisol levels, the loss of normal seasonal modulation is consistent with allostatic load—the physiological cost of maintaining stability under challenging conditions (Romero et al. 2009; Brown and Vosloo 2017; Romero and Beattie 2022). Such altered functioning could fundamentally shift how animals engage with their environments, potentially sustaining activated physiological states and impeding recovery even under favorable conditions (Clinchy et al. 2013; Shields et al. 2016; Karin et al. 2020). Given that these herds return to the same allotments annually, investigating possible cumulative physiological effects due to repeated wolf encounters should be a priority for future research with larger sample sizes. Such cumulative effects could have cascading consequences for prey population dynamics, particularly if repeated seasons of disrupted physiological regulation affect reproduction, immune function, or individual survival.

Unexposed herds followed typical seasonal patterns, with cortisol levels declining as temperatures increased (Alvarez and Johnson 1973; Lee et al. 1976; Ghassemi Nejad et al. 2021). The temperature–cortisol relationship we found may reflect the physiological demands of thermoregulation, as maintaining core body temperature in cold environments requires increased metabolic activity that can elevate circulating glucocorticoids (Bhimte et al. 2018). In managed cattle systems, colder temperatures also coincide with additional stressors including reduced forage availability, increased energy expenditure, and changes in social dynamics due to confined or supplemental feeding practices (Young 1981, 1983; Leng 1990; Munksgaard and Simonsen 1996; Hemsworth and Barnett 2000; Broom and Fraser 2007; Park et al. 2018; Wang et al. 2023). As such, summer grazing contexts may have contributed to overall cortisol reduction due to less handling, more space, and fewer social disruptions (Hemsworth and Barnett 2000). While the transition from winter grassland grazing to summer alpine grazing can initially elevate hair cortisol before stabilizing (Comin et al. 2011; Ghassemi Nejad et al. 2021), alpine grazing conditions may ultimately reduce cortisol relative to confined settings (Ghassemi Nejad et al. 2021). In contrast, wolf‐exposed herds showed an inverted response to temperature, with cortisol concentrations increasing rather than decreasing as temperatures rose. This suggests that wolf presence may interfere with normal seasonal cortisol modulation—potentially disrupting the temperature‐driven hormonal adjustments that typically occur in cattle under varying environmental conditions. Additionally, wolf exposure was treated as a binary variable based on whether herds grazed within the documented Lassen Wolf Pack home range; future studies incorporating continuous measures of predation risk intensity (e.g., proximity to den sites, frequency of wolf encounters) could reveal dose–response relationships. Non‐wolf predators (bears, mountain lions, coyotes) were present across all study areas and are not expected to vary systematically with wolf exposure, yet we cannot fully exclude unmeasured environmental heterogeneity as a contributing factor. Finally, given the small sample of wolf‐exposed herds—including one herd with only two sampled individuals—and uncertainty in effect magnitude, replication with larger samples is needed to confirm this pattern.

Our findings contribute to broader understanding of how recovering predators indirectly affect sympatric prey. As carnivores recolonize ecosystems, prey populations must reestablish physiological responses to predator presence that may have been lost or attenuated over generations of predator absence (Schweiger et al. 2019). Our findings suggest that this relearning process involves not simply elevated stress, but a fundamental restructuring of how prey physiologically respond to environmental cues. While extensive research examines direct predation effects, non‐consumptive effects of predators remain poorly quantified in many systems (Sheriff et al. 2011; Clinchy et al. 2013). The disrupted cortisol regulation we observed in wolf‐exposed herds suggests that predators can alter prey physiology through mechanisms independent of actual predation—a pathway that could significantly influence prey population dynamics. As carnivores recolonize western North America, understanding how predator presence reshapes prey physiological responses to environmental variation will be critical for predicting ecosystem‐level consequences of predator recovery (Stier et al. 2016; Wilmers et al. 2025). Future research should examine whether these physiological disruptions correlate with measurable fitness or population‐level consequences, and whether the mechanisms we document in cattle apply to wild prey species.

Beyond the ecological implications, understanding these physiological mechanisms has practical relevance for livestock production and welfare in regions where carnivores are recovering. The disrupted cortisol regulation we observed could potentially have consequences for cattle welfare and productivity. In livestock, sustained glucocorticoid elevation has been linked to reduced fertility, disrupted sleep, suppressed immunity, and lower milk yields (Dobson et al. 2001; Dahl et al. 2016; Cooke 2017). In cattle, stress‐induced glucocorticoid secretion disrupts follicular development and ovulation (Moberg 1991; Sheriff et al. 2011) while also impairing lactation by compromising the transfer of antibodies and nutrients through colostrum and milk (Brown and Vosloo 2017). In humans, elevated cortisol is associated with increased spontaneous abortion risk (Nepomnaschy et al. 2006). Whether the physiological patterns we document translate to measurable changes in cattle health and productivity requires further investigation.

Our study provides the first evidence of disrupted cortisol regulation in beef cattle exposed to gray wolf presence, measured through hair cortisol analysis. Our preliminary findings reveal important research gaps for understanding predator reintroduction ecology. First, whether disrupted cortisol regulation consistently emerges across larger samples of wolf‐exposed herds remains unknown. Second, the mechanisms underlying this disruption warrant investigation—does wolf presence directly trigger altered physiological responses, or do wolves alter cattle behavior and habitat use in ways that indirectly disrupt cortisol regulation? Third, understanding whether these physiological disruptions correlate with measurable consequences for cattle fitness (reproduction, survival, immune function) would strengthen links between physiology and population‐level outcomes. Finally, examining whether the mechanisms we document apply to wild prey species experiencing predator reintroduction would illuminate the generality of non‐consumptive predator effects across systems. As carnivore recovery programs expand across working landscapes globally, quantifying these indirect physiological mechanisms will be essential for predicting and managing ecosystem consequences of predator reintroduction.

## Author Contributions

**Christina M. Nord:** conceptualization (supporting), data curation (lead), formal analysis (lead), methodology (lead), visualization (lead), writing – original draft (lead), writing – review and editing (equal). **Alexander J. Pritchard:** conceptualization (supporting), formal analysis (supporting), methodology (supporting), visualization (equal), writing – review and editing (equal). **Rosemary A. Blersch:** conceptualization (supporting), data curation (supporting), formal analysis (supporting), methodology (supporting), visualization (equal), writing – review and editing (equal). **Brenda McCowan:** methodology (supporting), project administration (supporting), resources (supporting), supervision (equal), writing – review and editing (equal). **Jessica J. Vandeleest:** methodology (supporting), project administration (supporting), resources (supporting), supervision (equal), writing – review and editing (equal). **Kenneth W. Tate:** conceptualization (equal), funding acquisition (supporting), methodology (supporting), project administration (supporting), resources (supporting), supervision (equal), writing – review and editing (equal). **Tina L. Saitone:** conceptualization (equal), funding acquisition (lead), project administration (lead), resources (lead), supervision (equal), writing – review and editing (equal).

## Funding

This research was supported by a grant from the Western Sustainable Agriculture Research and Education (#SW22‐931) and the Russell L. Rustici Rangeland and Cattle Research Endowment.

## Ethics Statement

All research involving cattle was conducted in accordance with protocols approved by the University of California, Davis Institutional Animal Care and Use Committee (IACUC Protocol #22895). The study was carried out under the supervision of the UC Davis IACUC and followed all institutional guidelines for the ethical treatment of agricultural animals in research settings.

## Conflicts of Interest

The authors declare no conflicts of interest.

## Supporting information


**Data S1:** ece373431‐sup‐0001‐Supinfo.docx.

## Data Availability

Data and code used in this study are available from the Dryad Digital Repository https://doi.org/10.5061/dryad.s7h44j1mk, along with the description of the data.
