# Supplementary material for: Wolf Presence Disrupts Seasonal Variation in Hair Cortisol Among Free‐Ranging Beef Cattle
Source: Ecol Evol. 2026 Apr 6;16(4):e73431. doi: 10.1002/ece3.73431 (PMC13051829; doi:10.1002/ece3.73431)
Supplement: Supplementary file 1 — Data S1: ece373431‐sup‐0001‐Supinfo.docx. [file ECE3-16-e73431-s001.docx]

**Supplementary Materials
Wolf presence disrupts seasonal variation in hair cortisol among free-ranging beef cattle**

Christina M. Nord1,2*, Alexander J. Pritchard1,2, Rosemary A. Blersch1,2, Brenda McCowan1,2, Jessica J. Vandeleest1,2 , Kenneth W. Tate3, & Tina L. Saitone4

1 California National Primate Research Center, University of California Davis, Davis, CA, USA

2 Department of Population Health & Reproduction, School of Veterinary Medicine, University of California Davis, Davis, CA, USA

^3^Department of Plant Sciences, University of California Davis, Davis, CA, USA

^4^ Department of Agricultural and Resource Economics, University of California Davis, Davis, CA, USA

**S1. *Causal inference checks***

To clarify our assumptions about the relationship between wolf presence and cortisol concentrations, we constructed a directed acyclic graph (DAG) (Figure S1). Our primary exposure of interest was wolf presence, and our outcome was cortisol concentration. We included breed/color as an individual-level predictor of cortisol and herd as an adjusted variable. Season determines the location of animals (as cattle are moved to different grazing allotments seasonally), and location is represented as a latent (unobserved) variable. Location influences which herd occupies that allotment, whether wolves are present, and local temperature conditions. Season also influences temperature directly. Wolf presence, herd, and temperature are all assumed to affect cortisol. This causal structure highlights two key considerations. First, location is a potential confounder of the wolf presence–cortisol relationship but is not directly observed; its effects are mediated through observable variables including herd, wolf presence, and temperature. Second, temperature lies on pathways from both season and location to cortisol and therefore may act as a mediator. To properly estimate the causal effect of wolf presence on cortisol while adjusting for herd, our models must also include temperature to close remaining backdoor paths. Our model including both herd and temperature estimate the direct effect of wolf presence conditional on these variables.

A key advantage of the DAG framework is that it incorporates all variables believed to be causally relevant—including variables that were not or could not be measured (such as location)—allowing us to evaluate whether our measured variables are sufficient to identify causal effects while avoiding confounds (Pearl, 2009). Variables appearing in the DAG but not in the final statistical model either (1) lie on causal pathways we wish to estimate and thus should not be conditioned on to avoid blocking the effect of interest, or (2) are d-separated from the exposure-outcome relationship given our conditioning set—meaning they are conditionally independent of the outcome given our adjustment variables and do not require explicit inclusion (Textor et al., 2011). The DAG confirmed that conditioning on herd and temperature constitutes a sufficient adjustment set for estimating the effect of wolf presence on cortisol.


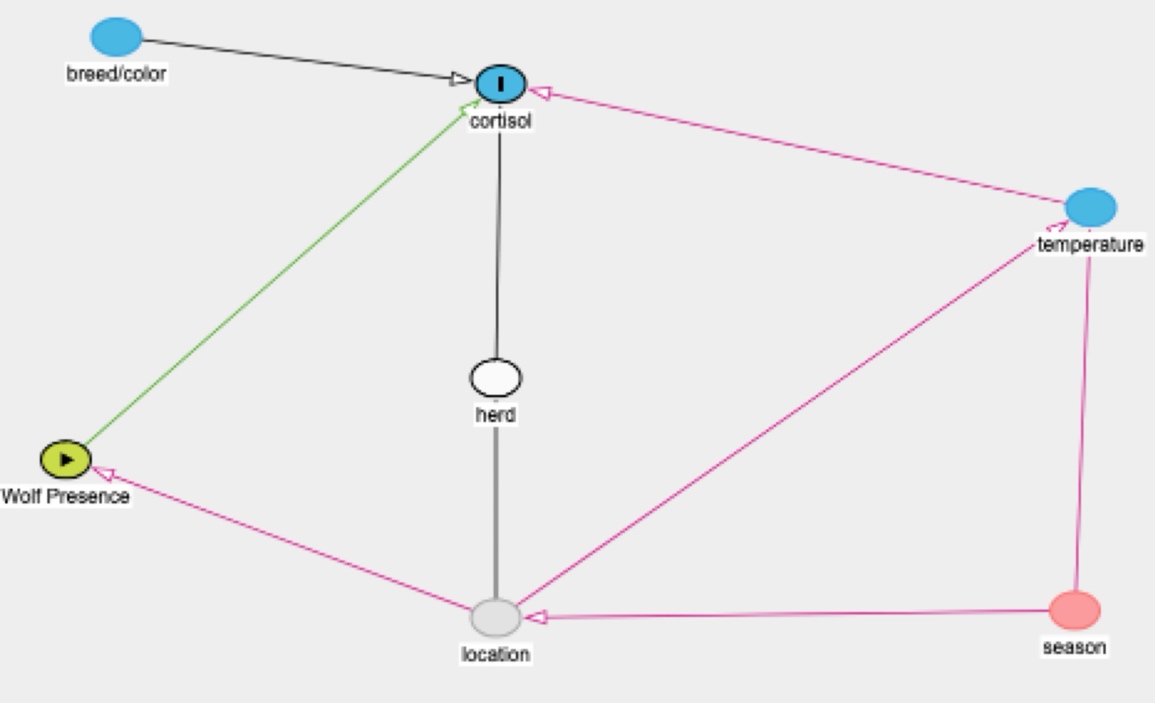


**Figure S1 Directed acyclic graph (DAG) representing the assumed causal relationships between wolf presence and cortisol concentrations.** Wolf presence (exposure) and cortisol (outcome) are highlighted, with breed/color included as an individual-level predictor of cortisol. Herd and season jointly determine location, which in turn influences wolf presence, local temperature, and cortisol. Season also affects temperature directly. Arrows indicate assumed causal effects, with temperature potentially acting as a mediator. This DAG illustrates potential confounding by location and guided the selection of adjustment variables in our model, clarifying whether estimates represent the total or direct effect of wolf presence on cortisol.


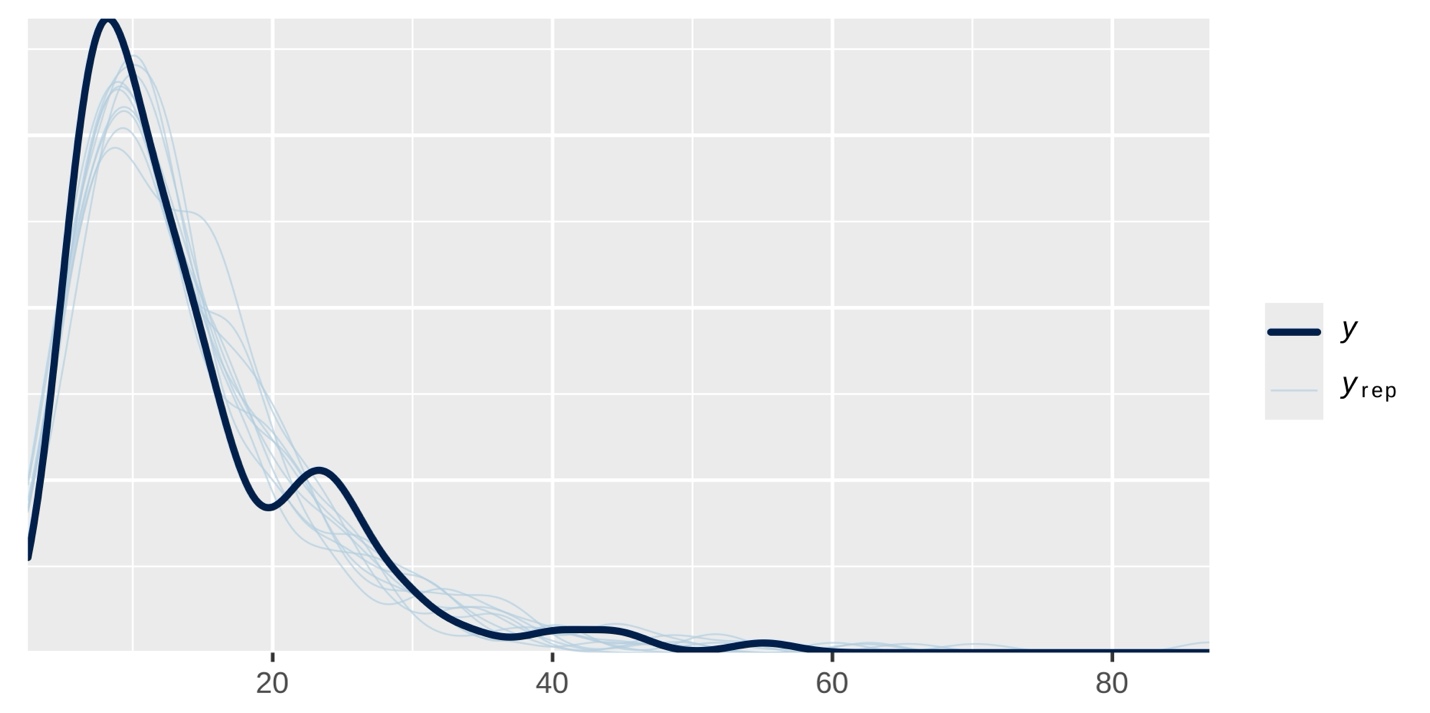


**Supplementary Figure 2** Posterior predictive check using a density overlay plot comparing observed values (y, dark blue line) to simulated datasets from the fitted model (y _rep_, light blue lines).


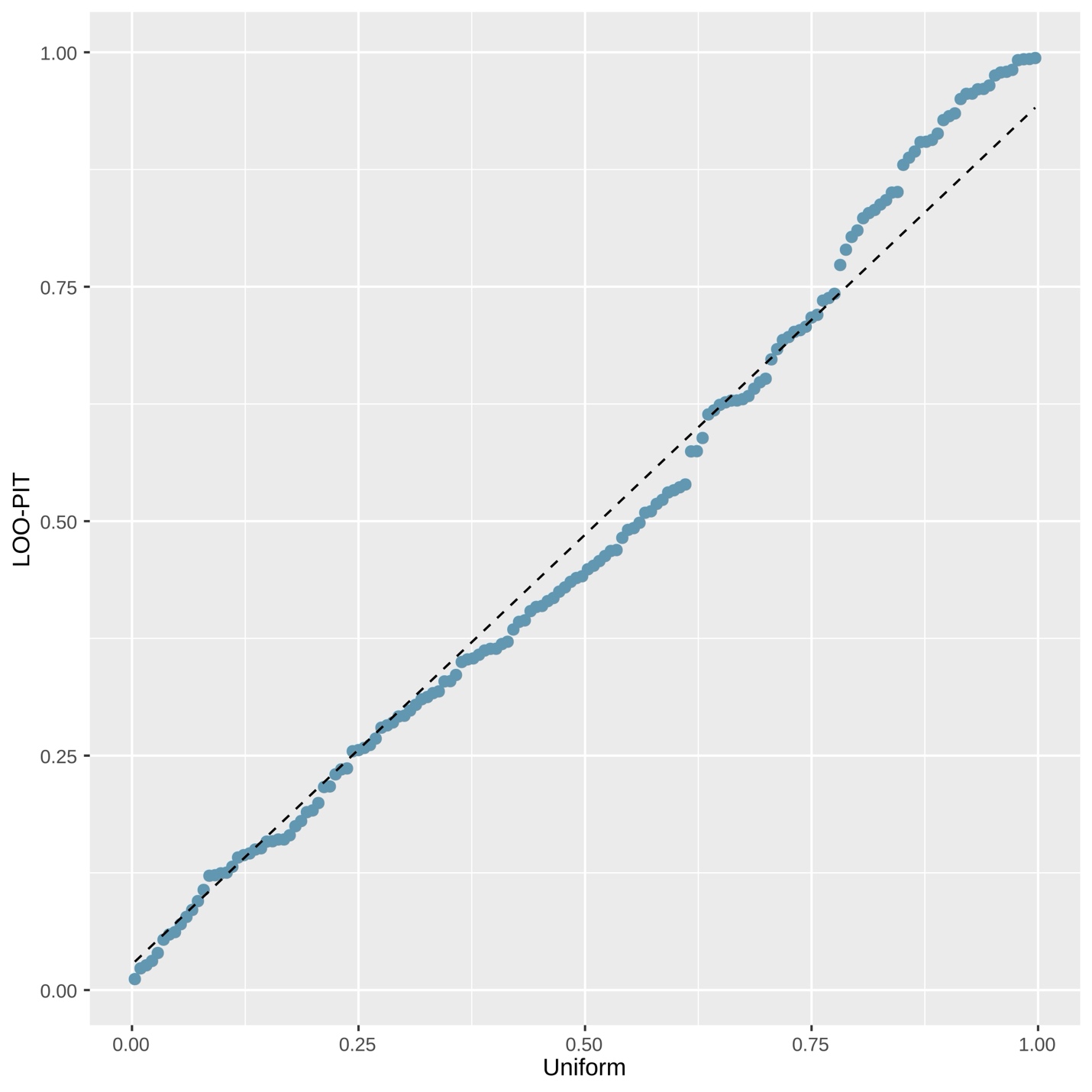


**Supplementary Figure 3** Leave one out probability integral transformation (LOO-PIT) posterior predictive check showing the empirical cumulative distribution function of LOO-PIT values (blue points) against the uniform distribution (dashed line).


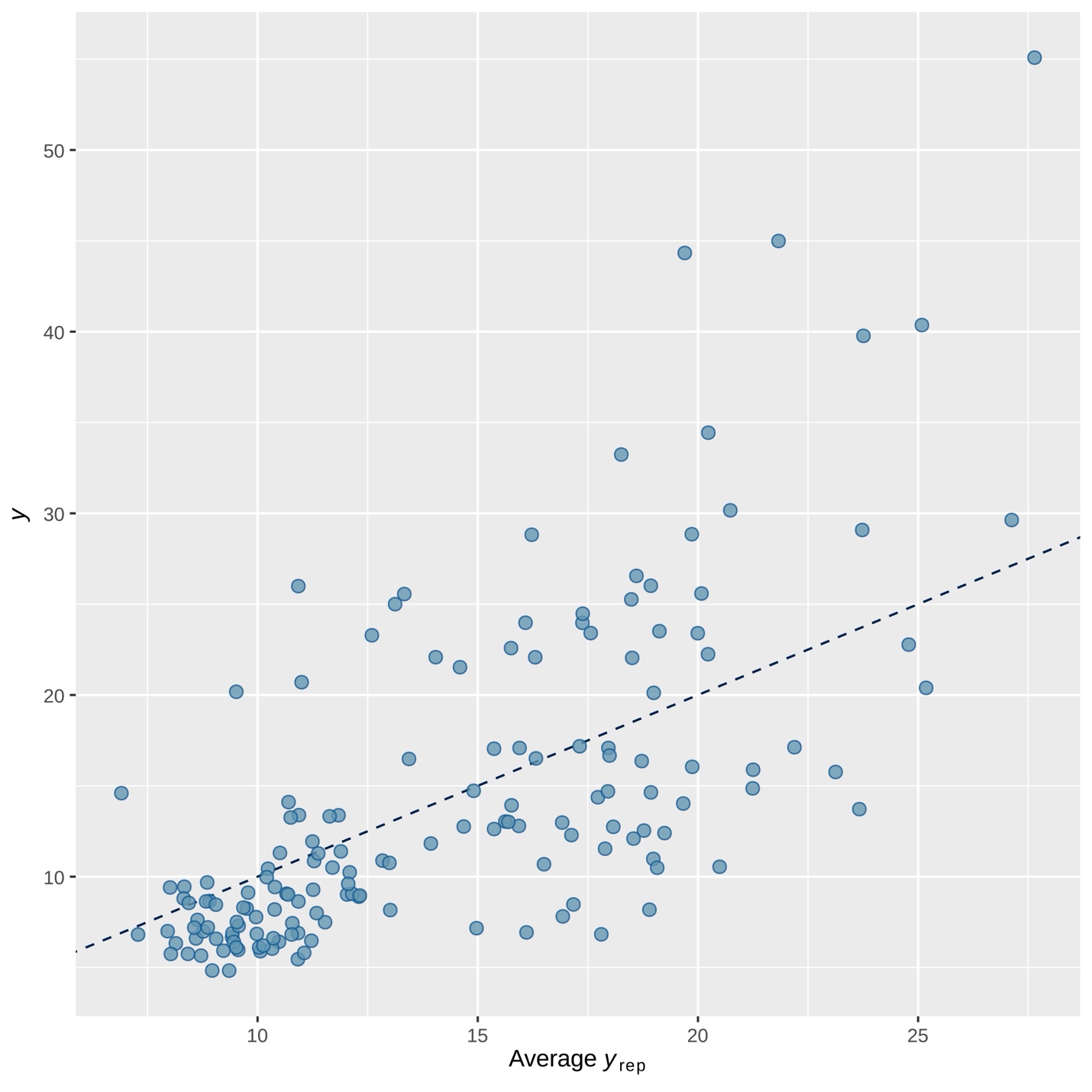


**Supplementary Figure 4** Posterior predictive check comparing observed test statistic (dashed line) to distribution of the same statistic computed from model simulations (points).


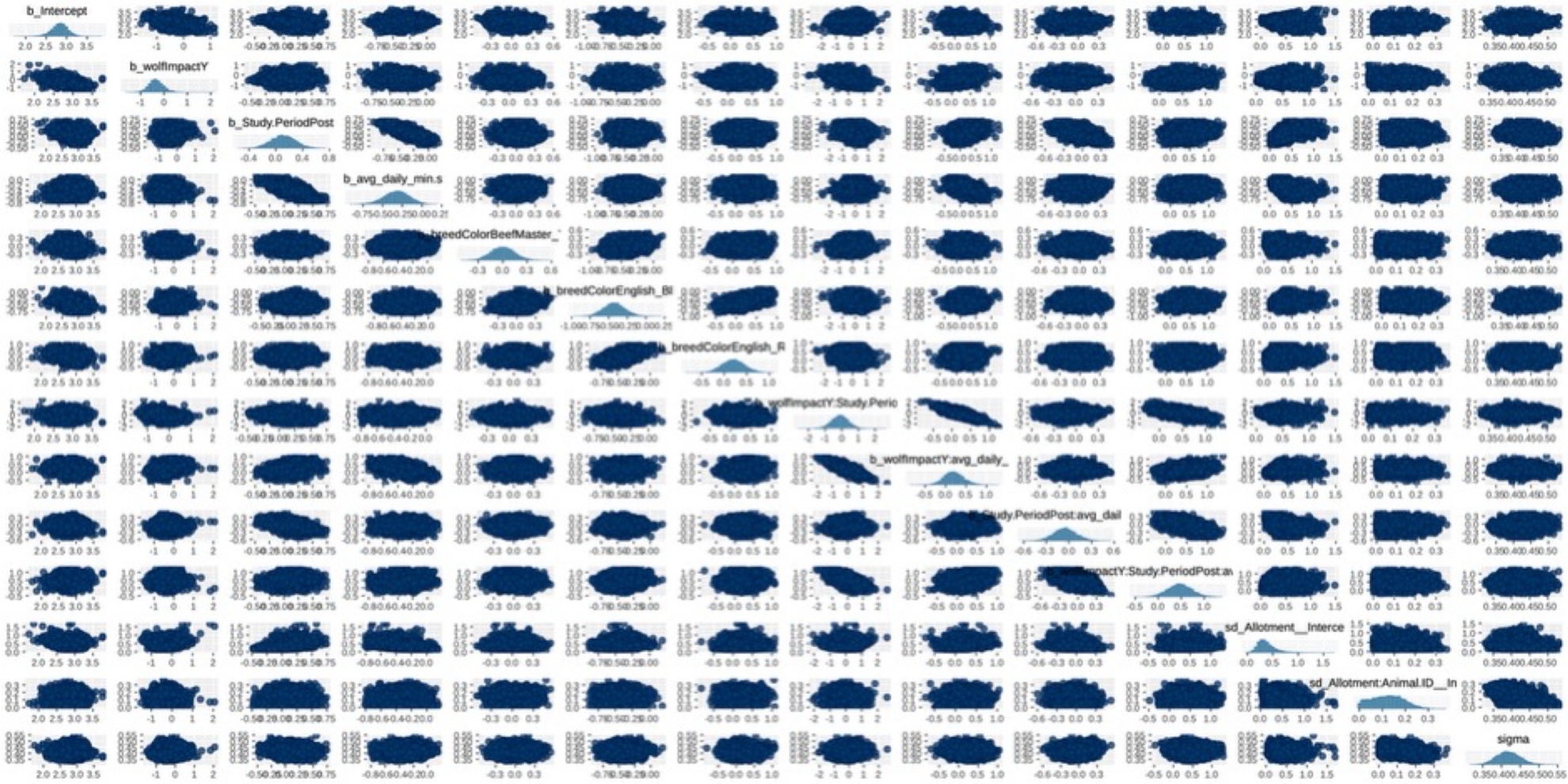


**Supplementary Figure 5** Pairs plot of posterior parameter samples showing correlations and marginal distributions. Each off-diagonal panel represents the joint posterior distribution of two parameters, with diagonal panels showing individual parameter densities.

**Table S1. Estimated Marginal Means and Contrasts of Hair Cortisol Concentrations**

*Part A: Estimated Marginal Means*

| **Wolf Exposure** | **Study Period** | **Temperature Level** | **Estimated Mean (pg/mg)** | **95% HPD Lower** | **95% HPD Upper** |
| --- | --- | --- | --- | --- | --- |
| No Wolves | Winter | Cooler (-1 SD) | 24.8 | 15.6 | 37.5 |
| Wolves | Winter | Cooler (-1 SD) | 14.3 | 7.6 | 23.7 |
| No Wolves | Summer | Cooler (-1 SD) | 28.7 | 11.4 | 59.0 |
| Wolves | Summer | Cooler (-1 SD) | 9.3 | 0.6 | 35.2 |
| No Wolves | Winter | Mean (0) | 18.2 | 12.2 | 25.6 |
| Wolves | Winter | Mean (0) | 12.7 | 6.5 | 21.3 |
| No Wolves | Summer | Mean (0) | 19.9 | 12.3 | 30.7 |
| Wolves | Summer | Mean (0) | 12.6 | 3.7 | 28.6 |
| No Wolves | Winter | Warmer (+1 SD) | 13.3 | 7.1 | 20.5 |
| Wolves | Winter | Warmer (+1 SD) | 11.3 | 3.8 | 22.7 |
| No Wolves | Summer | Warmer (+1 SD) | 14.0 | 8.5 | 19.4 |
| Wolves | Summer | Warmer (+1 SD) | 16.9 | 9.8 | 28.0 |

*Part B: Contrasts of Temperature Slopes by Wolf Exposure*

| **Study Period** | **Comparison** | **Temperature Contrast** | **Difference (pg/mg)** | **95% HPD Lower** | **95% HPD Upper** |
| --- | --- | --- | --- | --- | --- |
| Winter | No Wolf - Wolf | Cooler to Mean | 5.05 | -3.03 | 15.3 |
| Winter | No Wolf - Wolf | Cooler to Warmer | 8.53 | -4.88 | 24.8 |
| Winter | No Wolf - Wolf | Mean to Warmer | 3.46 | -2.06 | 10.1 |
| Summer | No Wolf - Wolf | Cooler to Mean | 10.83 | -3.62 | 37.5 |
| Summer | No Wolf - Wolf | Cooler to Warmer | 21.17 | -7.13 | 59.1 |
| Summer | No Wolf - Wolf | Mean to Warmer | 10.00 | -2.60 | 23.9 |

Note. Estimated marginal means and contrasts were calculated using the emmeans package in R. Point estimates are posterior medians. HPD = highest posterior density interval with 95% probability. Part A shows predicted hair cortisol concentrations for each combination of wolf exposure, study period, and temperature level. Part B shows contrasts testing differences in temperature slopes between wolf-exposed and unexposed herds. Positive contrast values indicate that unexposed herds have a steeper negative temperature slope (greater decrease with temperature) compared to wolf-exposed herds.
